# The Use of Apatinib in Treating Nonsmall-Cell Lung Cancer: Case Report and Review of Literature

**DOI:** 10.1097/MD.0000000000003598

**Published:** 2016-05-20

**Authors:** Lin Ding, Qing-Jian Li, Kai-Yun You, Zhi-Min Jiang, He-Rui Yao

**Affiliations:** From the Guangdong Provincial Key Laboratory of Malignant Tumor Epigenetics and Gene Regulation (LD, Q-JL, K-YY, Z-MJ, H-RY); and Department of Oncology (LD, Z-MJ, H-RY), Sun Yat-Sen Memorial Hospital of Sun Yat-Sen University, Guangzhou, China.

## Abstract

Apatinib is a novel tyrosine kinase inhibitor targeting vascular endothelial growth factor receptor-2, which has been proved to be effective and safe in treating heavily pretreated patients with gastric cancer.

The aim of the study was to explore the use of apatinib in treatment of nonsmall cell lung cancer and its side effects.

We report 2 patients presented with advanced nonsmall-cell lung cancer, who received apatinib after failure in the first- or third-line chemotherapy. They are treated with apatinib in daily dose of 850 mg, 28 days per cycle.

Favorable oncologic outcomes were achieved in the 2 cases after the treatment of apatinib. Patient I's progression-free-survival has increased to 4.6 months after palliative therapy of apatinib, whereas Patient II nearly 6 months. The common side effects of apatinib were hypertension and hand-foot syndrome; however, the toxicity of apatinib was controllable and tolerable.

Apatinib may be an option for advanced nonsmall cell lung cancer after failure of chemotherapy or other targeted therapy. But that still warrants further investigation in the prospective study.

## INTRODUCTION

Lung cancer ranks first in the mortality rates of cancers in China, and its incidence has been climbing up year by year. Nonsmall-cell lung cancer (NSCLC), which is usually in advanced stage when diagnosed, accounts for >70% of lung cancer.^1^ As we know, angiogenesis is a key process for cell growth, especially for the tumor growth.^2^ And the vascular epidermal growth factor (VEGF) can activate the downstream pathway to stimulate the proliferation of vessel endothelium via binding vascular epidermal growth factor receptor (VEGFR), thus leading to the growth of tumor. Studies have revealed that antiangiogenesis drugs inhibit the growth of solid tumors including NSCLC.^3^

As the first generation of oral antiangiogenesis drug created in China, apatinib which targets mainly at VEGFR-2 has a significant effect on the treatment of the advanced gastric carcinoma, significantly prolonging overall survival time (OS) of the advanced gastric cancer patients who failed in the second-line treatment. Apatinib has been known for its simplicity, compliance, and less side effects.^4^ Recently, more and more clinical practices are using apatinib in advanced metastatic gastric cancer and breast cancer. However, there is no report to evaluate its efficacy and safety in patient with nonsmall-cell lung cancer. Herein, the cases for the advanced metastatic NSCLC using Apatinib in our hospital are as follows.

### Case Presentation

**FIGURE 1:**
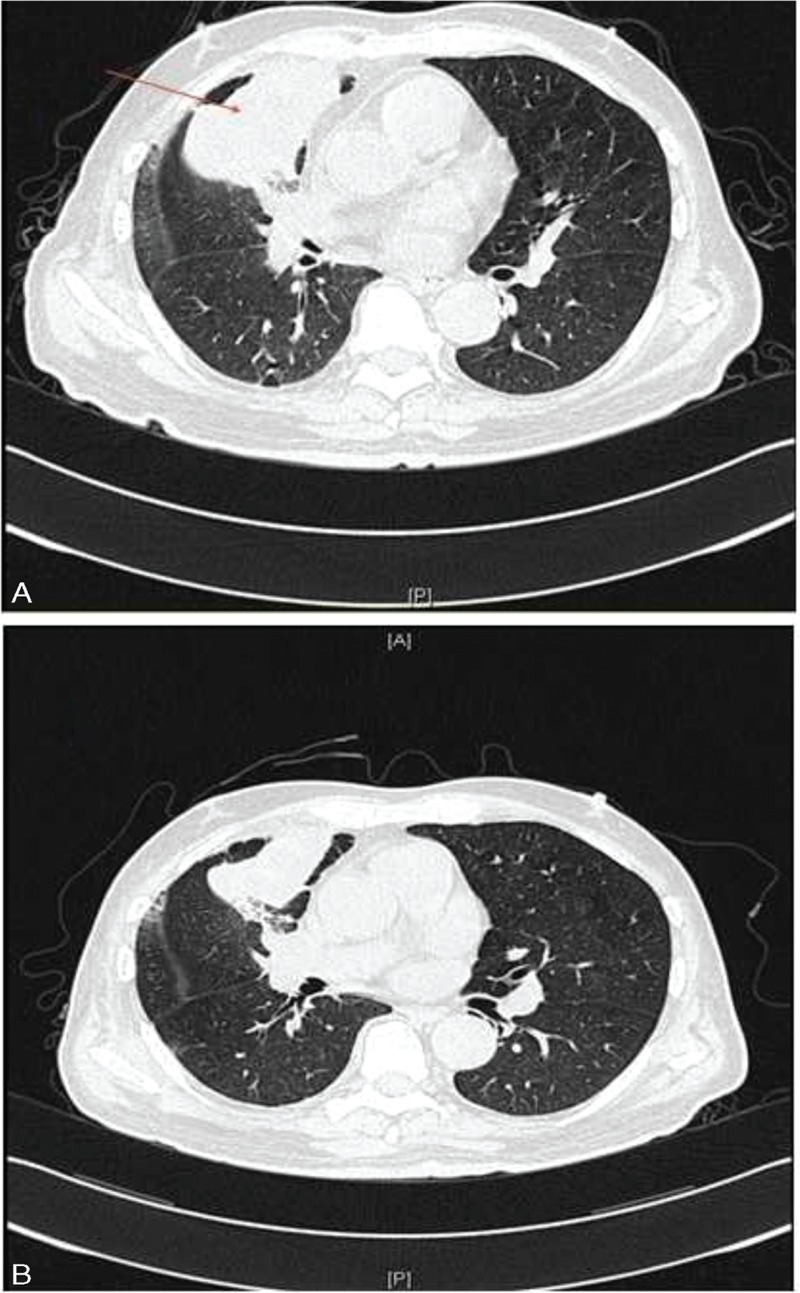
CT shows that mass in right upper lobe (A); mass is smaller after 3 month of apatinib treatment (B). CT = computed tomography.

Patient I, male, 70-year old, admitted to hospital on March 15, 2015, due to “recurring headache and dark stool defecation for 1 month.” Cranium MR indicated that there was a space-occupying lesion at the junction of parietal-occipital lobe, and malignant tumor could be considered. Chest and abdomen computed tomography (CT) scan showed that there was a lesion at the right upper lobe anterior segment, with multiple metastasis in the middle and low lobe of right lung and multiple lymph nodes metastasis in mediastinum and right hilus pulmonis. Both of the adrenal glands were also found to be with metastatic lesion. Gastroscope revealed that the mass on duodenum could be a metastatic tumor. The postoperative pathological result of the metastatic encephaloma palliative operation (March 25, 2015) indicated that it is poorly differentiated adenocarcinoma which originated from primary lung cancer. No gene mutations were detected in Anaplastic Lymphoma Kinase (ALK) or Epidermal Growth Factor Receptor (EGFR) examinations. The diagnosis was right lung adenocarcinoma with multiple metastases, which was treated by chemotherapy of docetaxel for 1 cycle (April 21, 2015). CT scan (May 5, 2014) indicated that compared with the previously one, the masses at the right upper lobe anterior segment and the ones in the middle lobe together with pulmonary atelectasis were bigger and more severe. The therapeutic evaluation was progressive disease (PD). Refusing second-line chemotherapy, the patient started oral administration of apatinib (850 mg/d) (May 28, 2015). After 1 month, CT scan (August 13, 2015) showed that therapeutic evaluation was stable disease (SD) and the mass reduced partially. Tumor indexes came down (Figure 1). Four months later, CT scan showed that therapeutic evaluation was PD. After taking apatinib, this case's progression-free-survival (PFS) has increased to 4.6 months.

**FIGURE 2:**
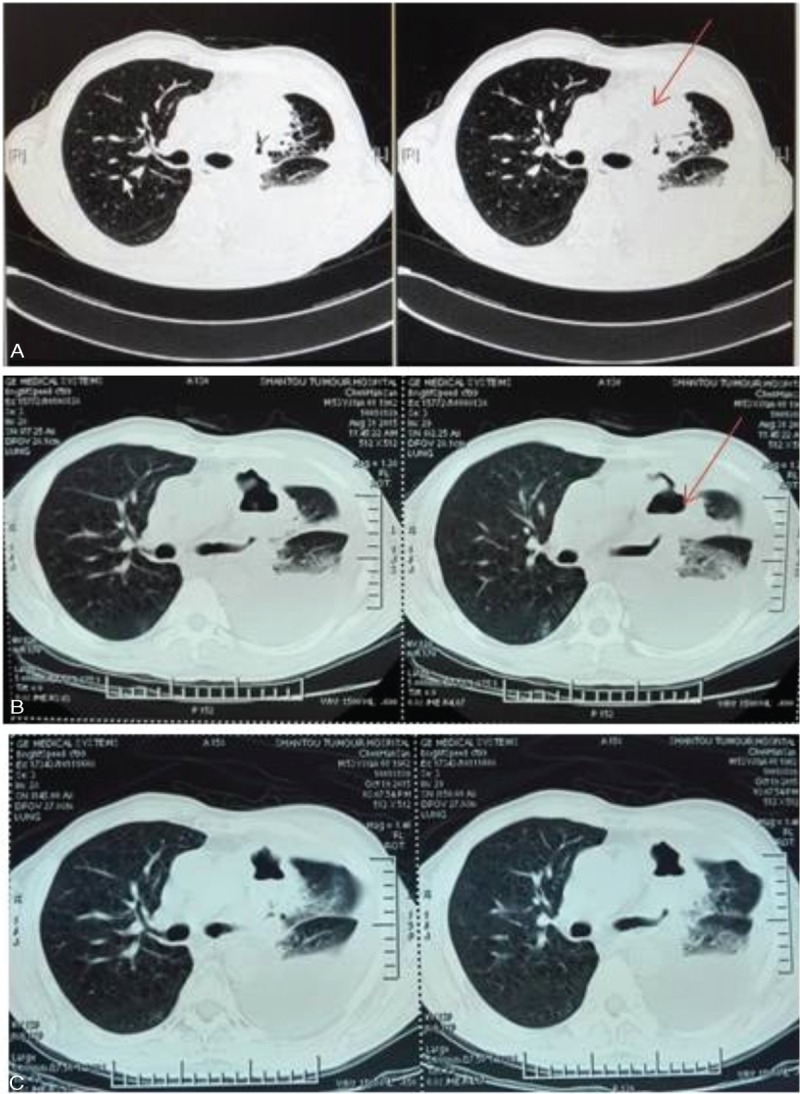
CT shows the mass in the lung (arrow indicate), (A) before apatinib treatment (July 2015). (B), (C) Apatinib treatment for 1 month and 3 months, respectively; local cavity can be seen. CT = computed tomography.

Patient II, male, 53-year-old, was examined by bronchofiberscope (Mar 2014) in local hospital due to left chest pain for more than a month. Pathological report revealed that it was squamous carcinoma on left upper lobe. The diagnosis of positron emission tomography-computed tomography (PET-CT) indicated left upper lung squamous carcinoma with stage T3N2M1a. No gene mutations were detected by EGFR examinations. With chemotherapy of “navelbine+cisplatinum” for 6 weeks (April 2014 to January 2015), the therapeutic evaluation was PD. It was then replaced by docetaxel as second-line chemotherapy that the therapeutic evaluation was still PD. Afterward Chinese medicine was used for treatment. Suffering chest pain with mild anhelation (April 2015), he admitted to our hospital (May 8, 2014). Chest CT revealed mass on left upper lobe anterior segment with lung cancer considered, and pleural effusion occurred at the left thoracic cavity. Chemotherapy of “gemcitabine + nedaplatin” was prescribed for 2 cycles (May 22, 2015 to June 19, 2015) as a third-line treatment, during which the blood platelet rose to 1260 × 10^9^/L. The result of bone marrow aspiration was normal, so we considered the increase of blood platelet was related to tumor. Chest CT (July 14, 2015) showed that compared with the previous one (May 8, 2015), the mass on left upper lobe anterior segment became bigger and pleural effusion on the left increased. Hence, therapeutic evaluation was PD. From July 25, 2015, he took apatinib orally (850 mg/d). Chest CT (August 31, 2015) indicated that the therapeutic evaluation was partial response (PR), and 3 months later chest CT indicated that therapeutic evaluation was SD (Figure 2), blood platelet decreased to the normal level. Until now, the patient has achieved a PFS of nearly 6 months.

## DISCUSSION

Studies revealed that new vessels provide nutrient and oxygen for tumors. By restraining angiogenesis, it is possible to inhibit the growth of tumor, development, and metastasis. VEGF/VEGFR is an important set of ligand and receptor affecting the angiogenesis, which is over expressed on the surface of various tumors. In recent years, researchers have been trying to restrict the growth of tumor by restraining the combination between VEGF and VEGFR to stop the activation of the downstream pathway.^3,5^ The targeted therapy of anti-VEGF/VEGFR includes reducing the concentration of activated and freed VEGF and cutting off the VEGFR signal system.

Traditionally, the standard regime for advanced NSCLC is chemotherapy of 2 drugs based on platinum. Many studies revealed that adding another chemotherapy drug into the above platinum-based regime would not benefit NSCLC patients. On the contrary, it would probably increase the drug toxicity.^6^ It brings up a point that chemotherapy alone has reached the plateau period.^7^ At the end of the 20th century, the status of angiogenesis in lung cancer has been confirmed by several studies. Some studies found out that the increase in the density of capillaries is closely related to the growth of tumor and poor prognosis.^8,9^ The emergence of antiangiogenesis drugs brings hope to patients with advanced NSCLC. Bevacizumab is the only antiangiogenesis drug approved by Food and Drug Administration to be used in the treatment of NSCLC.

Bevacizumab, a recombinant human monoclonal antibody, exerts an effect of antiangiogenesis by binding VEGF to block its biological function.^7^ ECOG4599 study randomly distributes 878 patients who suffer recurring or advanced NSCLC (stage IIIB or IV) in 2 groups, paclitaxel + carboplatin group and chemotherapy + bevacizumab group. The results show that the overall survival (OS) in the chemotherapy + bevacizumab group is longer than that in the paclitaxel + carboplatin group (12.3 months vs 10.3 months, HR: 0.79, *P* = 0.003). The median progression-free survival (PFS) is 6.2 months and 4.5 months respectively (HR: 0.66, *P* < 0.001).^10^ Avail study also confirmed that chemotherapy in combination with bevacizumab benefits NSCLC patients. Avail is a Phase III randomized controlled study, which compares the treatment of gemcitabine and cisplatinum with different dosages of bevacizumab (7.5 g/kg or 15 g/kg) or placebo. It enrolls 1043 advanced NSCLC patients and the results shows longer PFS in patients treated with gemcitabine and cisplatinum regimes combined with both dosages of bevacizumab than placebo.^11^ Bevacizumab is the first and the only targeted drugs used in the first-line treatment of NSCLC and may prolong the OS in combination with chemotherapy so far.^7^

Apatinib is the first generation of oral antiangiogenesis drug invented in China, which mainly targets at VEGFR-2 as well as the receptor tyrosine kinase (RTK) such as c-kit, RET, and c-src.^12^ It was found that VEGF-2 promoted endothelial proliferation by activating the mitogen-activated protein kinase (MAPK) signaling pathway during the process of angiogenesis.^13^ And by blocking VEGFR-2, apatinib can suppress endothelial proliferation and finally lead to antiangiogenesis, which was confirmed to exerted an antitumor effect on various cancers.^14^

A study has revealed that apatinib showed significant competence in treating solid tumor. In the study, 45 patients with measurable tumors originated from lungs, gastrointestinal tract, and other organs were randomly assigned to the oral apatinib groups of 250 mg/d, 500 mg/d, 750 mg/d, 800 mg/d, and 1000 mg/d. After taking in apatinib, 7 patients achieved PR (18.9%) and 24 were with SD (64.9%).^15^ A Phase III, multicenter, open, and single arm clinical study included 38 metastatic breast cancer patients who suffered from failure of chemotherapy. All the patients were treated with apatinib (500 mg/d). Results revealed that the median PFS of 38 patients was 4 months (95% CI, 2.8–5.2 months), the median overall survival (OS) was 10.1 months (95% CI, 9.1–11.6 months). After the analysis of 36 patients with data available, the objective response rate (ORR) and disease control rate (DCR) were 16.7% (6/36) and 66.7% (24/36), respectively.^16^ Upon the approval by China Food And Drug Administration, apatinib has been suggested to treat advanced gastric adenocarcinoma and adenocarcinoma in the gastroesophageal junction in China. This guideline is mainly based on 2 clinical trials. One is a Phase III randomized controlled study in which 267 advanced gastric cancer patients who failed in the second-line chemotherapy were included. There were 176 patients who were assigned to the apatinib group, whereas the other 91 patients were into the placebo group. It was found that the OS of apatinib group was significantly longer than the placebo group (195 days vs 140 days, HR = 0.71, *P* < 0.016). Median PFS were 78 days and 59 days in the apatinib group and placebo group, respectively (HR: 0.44, *P* < 0.0001). And the PFS was significantly also longer in the apatinib group.^17^ Another study included 141 advanced gastric cancer patients who failed in the second-line chemotherapy. The patients were randomized into the placebo group or 2 apatinib groups with daily dose of either 850 mg or 425 mg. Median OS for the 3 groups were 2.50 months, 4.83 months, and 4.27 months, respectively. Median PFS were 1.40 months, 3.67 months, and 3.20 months, respectively. Significant differences were found in both OS and DFS among the groups.^18^ To explore the further use of apatinib, a phase II, multicenter, placebo-controlled trial recruited 135 advanced nonsquamous NSCLC patients who failed over 2 lines of treatment. The patients received apatinib (750 mg/d) or placebo with allocation ratio of 2:1. The end point was the disease progression or unacceptable toxicity. The results showed that median PFS, response rate, and disease control rate in the apatinib group were all better than the placebo group (4.1 months vs 1.9 months, *P* < 0.0001; 12.2% vs 0%, *P* = 0.0158; 68.9% vs 24.4%, *P* < 0.0001, respectively). The most frequent adverse effects were hypertension, proteinuria, and hand-foot syndrome, all of which were manageable.^19^

The reasons for the use of apatinib in the 2 cases in our present study were due to intolerant side effects of chemotherapy or unsatisfactory treatment efficacy, but patients still have strong wish to continue treatment even after the failure of third-line chemotherapy. According to the general condition of patients, we treated them with apatinib in daily dose of 850 mg, 28 days per cycle. After a period of treatment, the disease was well controlled for a certain time. Although the 2 cases mentioned above were individual, apatinib did show its curative effect in nonsmall-cell lung cancer.

The common side effects of apatinib were hypertension and hand-foot syndrome; however, the toxicity of apatinib was controllable and tolerable.^17^ And it was suggested that the dose of 750 mg/d was relatively safe and well-tolerated.^15^ In our cases, hand-foot syndrome with grade 2 occurred and it was well managed. What is noteworthy is that 1 patient with squamous carcinoma benefited from apatinib.

It has always been a hot issue when it comes to treating cancer with combination of targeted drug and chemotherapeutic drugs. In view of the effects of other antiangiogenesis on advanced NSCLC, we can boldly think of 3 questions: (1) Whether the combination of apatinib and standard chemotherapy can improve the benefit in advanced NSCLC patients? (2) Whether apatinib can be another reliable targeted drug for lung cancer without EGFR gene mutation? (3) Whether apatinib can break the deadlock that bevacizumab is not supposed to be used in squamous cell lung carcinoma? To answer these questions, more clinical studies are needed and these questions can further offer guidelines in the clinical treatment.
